# Comparison of fracture rates between indigenous and non-indigenous populations: a systematic review protocol

**DOI:** 10.1136/bmjopen-2016-012124

**Published:** 2016-08-25

**Authors:** Sharon L Brennan-Olsen, Shae E Quirk, William D Leslie, Maree Toombs, Kara L Holloway, Sarah M Hosking, Julie A Pasco, Brianna J Doolan, Richard S Page, Lana J Williams

**Affiliations:** 1 Deakin University, Geelong, Victoria, Australia; 2 Institute for Health and Ageing, Australian Catholic University, Melbourne, Victoria, Australia; 3 Australian Institute for Musculoskeletal Science, The University of Melbourne, St Albans, Victoria, Australia; 4 Department of Medicine, University of Manitoba, Winnipeg, Manitoba, Canada; 5 Rural Clinical School, School of Medicine, University of Queensland, Toowoomba, Queensland, Australia

**Keywords:** Indigenous, fracture, Musculoskeletal

## Abstract

**Introduction:**

Over recent years, there has been concerted effort to ‘close the gap’ in the disproportionately reduced life expectancy and increased morbidity experienced by indigenous compared to non-indigenous persons. Specific to musculoskeletal health, some data suggest that indigenous peoples have a higher risk of sustaining a fracture compared to non-indigenous peoples. This creates an imperative to identify factors that could explain differences in fracture rates. This protocol presents our aim to conduct a systematic review, first, to determine whether differences in fracture rates exist for indigenous versus non-indigenous persons and, second, to identify any risk factors that might explain these differences.

**Methods and analysis:**

We will conduct a systematic search of PubMed, OVID, MEDLINE, CINAHL and EMBASE to identify articles that compare all-cause fracture rates at any skeletal site between indigenous and non-indigenous persons of any age. Eligibility of studies will be determined by 2 independent reviewers. Studies will be assessed for methodological quality using a previously published process. We will conduct a meta-analysis and use established statistical methods to identify and control for heterogeneity where appropriate. Should heterogeneity prevents numerical syntheses, we will undertake a best-evidence analysis to determine the level of evidence for differences in fracture between indigenous and non-indigenous persons.

**Ethics and dissemination:**

This systematic review will use published data; thus, ethical permissions are not required. In addition to peer-reviewed publication, findings will be presented at (inter)national conferences, disseminated electronically and in print, and will be made available to key country-specific decision-makers with authority for indigenous health.

**Strengths and limitations of this study**

- Our systematic review will fill a gap in the evidence-base by providing a comprehensive assessment of the existing literature to compare fracture rates between indigenous and non-indigenous populations worldwide. In reviewing the literature, we will establish (1) if a difference exists, (2) the magnitude of any differences and (3) what risk factors for differences in fracture rates have been identified.
- Study selection, data extraction and assessment of methodology will be conducted independently by two authors.
- The findings of this systematic review will inform the evidence-base that decision-makers working in health policy can use to enhance the targeting of interventions to prevent fractures in indigenous persons.
- A potential limitation of this review might be that different countries have different definitions of indigenous status; however, to address this, we will include articles that align with Article 33 of the *United Nations Declaration on the Rights of Indigenous Peoples* definition of indigenous status, where the importance of self-identification as an indigenous person is underlined.
- It is possible that we may identify a paucity of data in this area of enquiry; this may reflect systemic disenfranchisement from the mainstream research community and thus identify a research gap, which could be considered analogous to the postfracture ‘care gap’.

## Introduction

According to the United Nations, there are >370 million indigenous persons worldwide living in ∼70 countries. By definition, indigenous persons are the descendants of those who originally inhabited a country or geographical region and are spread across the world from the Arctic to the South Pacific. Among indigenous peoples are those of the Americas (eg, the Native Americans in the USA, the First Nations and Métis of Canada, the Mayas in Guatemala and the Aymaras in Bolivia), the Inuit and Aleutians of the north circumpolar region, the Saami of northern Europe, the Aborigines and Torres Strait Islanders of Australia and the Maori of New Zealand. Indigenous persons practice unique traditions and retain social, cultural, economic and political characteristics that are distinct from those of the dominant societies in which they live.^1^ Indigenous peoples constitute ∼5% of the world's population; however, they account for ∼15% of the world's poor.^2^

Among high-income countries, four countries are often considered comparable in terms of indigenous well-being: Australia, the USA, New Zealand and Canada. These four countries consistently place near the top of the United Nations Development Programme's Human Development Index rankings, yet all have minority indigenous populations with much poorer health and social conditions than their non-indigenous compatriots.^3^ Disproportionately reduced life expectancies are observed for indigenous peoples compared to non-indigenous peoples;^3^ ^4^ in Australia, there is a ∼10-year gap with the average life expectancy being 59.5 years,^5^ ^6^ American Native peoples and Alaskan natives live an average of 70.8 years, New Zealand Maoris live an average of 71.1 years, and Canadian First Nations, Métis and Inuit peoples live an average of 72.8 years.^3^ ^5^ Although the United Nations advocates for the right of everyone to the highest attainable standard of health,^7^ Canadian First Nations women have lower health-related quality of life than Caucasian women.^8^ Indeed, data suggest that 80% of the life expectancy gap between indigenous and non-indigenous persons is attributable to chronic diseases.^9^

It is well documented that a fragility fracture due to osteoporosis is a strong independent risk factor for subsequent fracture.^10^ ^11^ Furthermore, a fracture of the hip can reduce life expectancy^12^ ^13^ and quality of life, and places a greater demand on health infrastructure.^13–15^ Importantly, data from Manitoba, Canada, showed not only an independent contribution of First Nations status to postfracture mortality but also a larger absolute increase in postfracture mortality for First Nations compared to non-First Nations peoples.^16^ Excess mortality attributable to fracture in indigenous persons has not been investigated in other countries. Furthermore, disability related to musculoskeletal diseases is increasing due to population growth and a shift in the population age structure.^17^ Explanatory variables that may account for the increased risk of fracture in indigenous persons are imperative to elucidate. For instance, we know that there is a heavy burden of chronic health conditions suffered by indigenous persons,^5^ ^18^ including type 2 diabetes.^19^ Thus, an improved understanding regarding potentially modifiable factors can enhance attempts to close the gap in life expectancy.

This article presents the protocol for a systematic review, which first aims to identify and compare all-cause fracture rates in indigenous compared with non-indigenous populations; this important area of enquiry has not been addressed by a comprehensive review of existing literature, as identified by a search of the Cochrane Library (performed 29 February 2016). Possible risk factors that contribute to any observed differences in fracture rates will also be investigated, as a secondary aim of interest. This protocol adheres to the Preferred Reporting Items for Systematic reviews and Meta-Analyses Protocols (PRISMA-P) guidelines.^20^

### Objectives

This systematic review will:

1. Identify published studies that compare all-cause fracture rates between indigenous and non-indigenous populations across the entire age spectrum;
2. Evaluate the methodological quality of all eligible studies according to a previously employed scoring system;^21^ ^22^
3. Analyse the level of evidence for all studies combined, and conduct two subgroup analyses to:
  - Compare high versus low methodological quality (above the median) to determine whether any bias is observed;
  - Explore heterogeneity by excluding those studies that did not account for socioeconomic variables.

## Methods

### Indigenous status

Rather than employing a definition of indigenous status, our inclusion criteria for eligible studies will align with Article 33 of the *United Nations Declaration on the Rights of Indigenous Peoples*, in which the importance of self-identification as an indigenous person is underlined.^1^ ^23^ However, we will also include articles that determine indigenous status by the use of country-specific identity registration systems.

### Criteria for considering studies for this review

The criteria for inclusion in this review will be: full-text articles that are epidemiological cohort, case–control and/or cross-sectional studies and that examine fracture rates in indigenous populations, or indigenous versus non-indigenous populations, inclusive of any country, sex or age.

Grey literature and conference presentations will be excluded. Furthermore, given that the purpose of this review is to ascertain whether bone quality and/or fracture rates differ between non-indigenous and indigenous populations, randomised controlled trials (RCTs) will be excluded. However, where possible, we will include baseline data from RCTs that pertain to fracture rates prior to intervention, as it is possible that the data from cases may provide relevant cross-sectional information, and data from the controls would be equivalent to a cohort study. Articles that investigate indigenous and non-indigenous persons but do not present findings separately for each group will not be eligible for inclusion.

### Search strategy and data extraction

We will perform a computer-generated search strategy using databases for medical, health and social sciences (PubMed, OVID, MEDLINE, CINAHL and EMBASE) to identify relevant literature, with no limits set on the date of publication. We will apply the following medical subject headings (MeSH): ‘osteoporosis’ OR ‘fractures’ OR ‘bone’ AND (‘Indigenous’ OR ‘Aborigines’ OR ‘Inuit’ OR ‘Indians, Central American’ OR ‘Indians, North American’ OR ‘Indians, South American’ OR ‘Oceanic Ancestry Group’). Key words (informed by the *United Nations Permanent Forum on Indigenous Issues*^1^ ^23^) will include: aboriginal, Aleutians, American Indian, First Nation, Lakota, Maasai, Maori, Mayas, Métis, Native Americans, Native-born, Saami, Torres Strait Islander and indigenous peoples. Relevant truncation will be used for each database. Reference lists of relevant studies that fulfil the eligibility criteria will be independently hand-searched by two reviewers (SLB-O and BJD). One reviewer will perform the search strategy (SLB-O), and two further reviewers (SEQ and BJD) will confirm the selection of articles. Where articles are written in a language other than English, we will seek professional assistance with interpretation for a comparison against the selection criteria.

### Assessment of methodological quality of included articles

**Figure 1:**
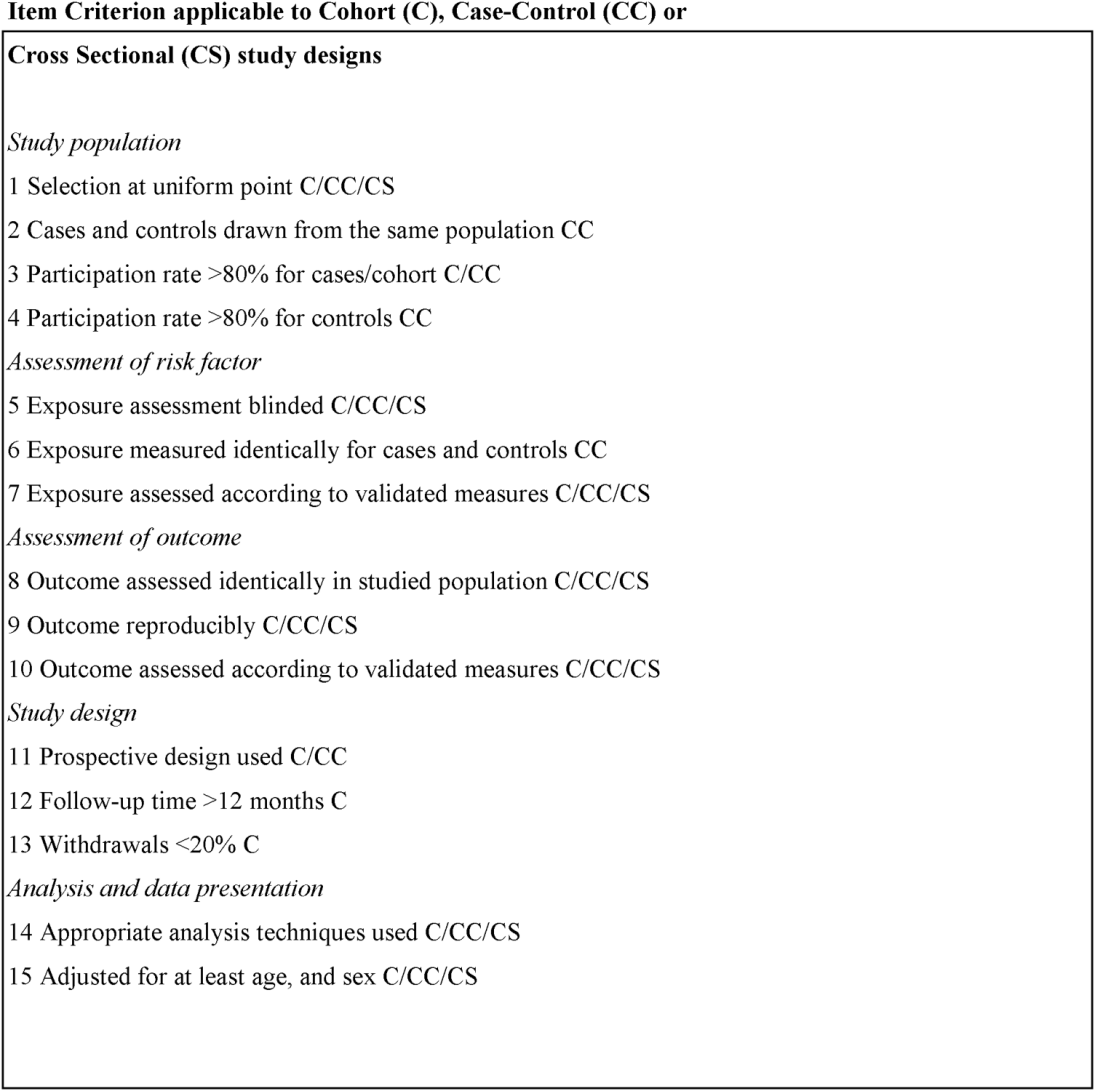
Criteria list for the assessment of methodological quality, modified from Lievense *et al*.^21^ ^22^

Data extracted from included studies will be analysed using a modified version of the methodological scoring system of Lievense *et al*.^21^ ^22^ We have previously employed this methodology to determine the quality of cohort, case–control and cross-sectional study designs in the musculoskeletal research field.^24–26^ As part of the modification to the Lievense *et al* tool, only those 15 criteria that assessed ‘internal validity’ of the studies were retained, while those 4 criteria that assessed ‘informativeness’ were removed. Briefly, the methodology of eligible studies will be scored using predetermined criteria as follows: positive (1) or negative (0), with 100% representing a maximum obtainable score for each of the study designs (figure 1). According to the scoring system, cohort studies reflect the optimal study design due to the inherent prospective qualities, followed by case–control studies and then cross-sectional study designs.

Two reviewers (SLB-O and SEQ) will independently score eligible studies using the Lievense *et al*^21^ ^22^ scoring system. Should the scorers disagree with differences that cannot be reconciled, a third reviewer will provide the final judgement in one consensus meeting (LJW). The total score (%) for each of the studies will be calculated and, subsequently, deemed as high quality if scoring above the median of the total scores.

### Presenting and reporting results

Results of this systematic review will be presented according to the PRISMA-P reporting guidelines.^20^ We will provide an adapted QUORUM diagram,^27^ which will present a flow chart of study selection, and reasons for including or excluding articles, including observational studies. A description of all relevant studies will be presented, and the following key information will be manually extracted from those articles eligible for inclusion: author, country from where the sample was drawn (including region/state/district where available), population description, indigenous status, fracture ascertainment and skeletal site. Results of the methodological scoring for all articles will be presented as a percentage. We will also provide details of the modelling procedures employed by each of the studies to investigate associations between indigenous status and fracture, including the factors that were included in models (including the exposure variable/s), the statistical results and a summary of the authors' findings.

We will conduct a meta-analysis and use statistical methods to control for heterogeneity where appropriate. We will also perform subset and/or sensitivity analyses to further explore heterogeneity, whereby those studies that did not account for socioeconomic variables are excluded. Should statistical heterogeneity prevents a numerical synthesis, we will employ a best-evidence synthesis to assess the level of evidence available, whereby the level of evidence could range from strong evidence to no evidence (table 1).^21^ ^22^ We have previously published best-evidence syntheses regarding determinants of fracture.^24^ ^26^

**Table 1:** Criteria for ascertainment of evidence level for best-evidence synthesis, adapted from Lievense *et al*^21^

Level of evidence	Criteria for inclusion in best-evidence synthesis
Strong evidence	Generally consistent findings in: Multiple high-quality cohort studies
Moderate evidence	Generally consistent findings in: One high-quality cohort study and >2 high-quality case–control studies >3 high-quality case–control studies
Limited evidence	Generally consistent findings in: Single cohort study One or two case–control studies or Multiple cross-sectional studies
Conflicting evidence	Inconsistent findings in <75% of the studies
No evidence	No studies could be found

### Dissemination

Findings from this systematic review will be published in a peer-reviewed scientific journal. Our results will also be presented at national and international conferences and will be made available to key country-specific decision-makers with authority for indigenous health.

### Ethics

Given that this systematic review will use published data, we do not require ethical permissions. However, our research processes will adhere to ethical and governance standards with regards to data management, and the presentation and discussion of our findings. Furthermore, our protocol has been informed by indigenous person/s from Australia, who is also engaged in our authorship list.

### Conclusion

To the best of our knowledge, this will be the first review of its kind to explore the association between indigenous status and fracture rates. Investigating whether differences exist in fracture rates between indigenous and non-indigenous populations has broad policy and practice implications. Furthermore, these data will inform the evidence-base to support existing and future health campaigns and resource allocation to improve health of indigenous peoples and reduce gaps in their life expectancy related to postfracture mortality. Should a paucity of data be identified, this will indicate a clear research gap, and a possible disenfranchisement from mainstream research, that will require urgent attention; however, the authors are aware of a number of studies that fulfil the eligibility criteria^28–32^ and, thus, will avoid the scenario of an ‘empty’ systematic review report.
